# CRISPR/Cas9‐targeted mutagenesis of the *OsROS1* gene induces pollen and embryo sac defects in rice

**DOI:** 10.1111/pbi.13388

**Published:** 2020-05-10

**Authors:** Yang Xu, Fangquan Wang, Zhihui Chen, Jun Wang, Wenqi Li, Fangjun Fan, Yajun Tao, Yanjie Jiang, Qian‐Hao Zhu, Jie Yang

**Affiliations:** ^1^ Institute of Food Crops Jiangsu Academy of Agricultural Sciences/Nanjing Branch of Chinese National Center for Rice Improvement Nanjing China; ^2^ Jiangsu Co‐Innovation Center for Modern Production Technology of Grain Crops Yangzhou University Yangzhou China; ^3^ CSIRO Agriculture and Food Canberra ACT Australia; ^4^ Institute of Life Science Jiangsu University Zhenjiang China

**Keywords:** rice, CRISPR/Cas9, *OsROS1*, sterility, pollen, embryo sac

Rice yield and sustainable production are important issues for global food safety (Itoh *et al.*, 2005). Sterility mutants are appropriate materials for understanding the molecular mechanisms underlying fertility regulation in rice, and are potential germplasm for production of hybrid seeds. The availability of the rice whole genome sequence enabled fine mapping and cloning of the key genes underlying the sterility trait and shed insights into the development of male and female gametophytes in the past decade, but our understanding on the genetic and molecular mechanisms underlying fertility remains limited.

*REPRESSOR OF SILENCING 1 (ROS1)* encodes a bi‐functional DNA demethylase that removes 5‐methylcytosine and nicks double‐stranded DNA (Gong *et al.*, 2002; Tang *et al.*, 2016). It was found that *OsROS1a*, a rice homolog of *Arabidopsis ROS1*, is indispensable for the development of gametophytes (Ono *et al.*, 2012). A recent study reported that accumulation of an alternatively spliced *ROS1* transcript reduced seed‐setting by 8.2% and produced seeds with multiple layers of aleurone (Liu *et al.*, 2018). CRISPR/Cas9 gene editing has been proved to be a powerful tool to generate knockout mutants for characterization of gene function in plants (Chen *et al.*, 2019). In this study, we create a number of *OsROS1* knockout mutants using CRISPR/Cas9 and further explored the role of *OsROS1* in regulation of rice fertility.

Two 20‐bp guide RNAs specific to *OsROS1* (*LOC_Os01g11900*) targeting the first (targeted site 1, TS1) and the fifteenth exons (targeted site 2, TS2) (Figure 1a) were designed, and used to generate the knockout vectors pOsCas9‐TS1 and pOsCas9‐TS2. Each vector was individually introduced into the rice cultivar Zhennuo19 by *Agrobacterium* transformation. For each target site, we identified seven mutated transgenics. Four lines were homozygous mutants; the remaining lines were bi‐allelic mutants (Figure 1b). Most of the mutations caused frameshifts in the coding region, giving rise to prematurely terminated proteins, and two edited lines (line 1‐3‐3 and 2‐4‐7) contained amino acid deletions and/or substitutions. Furthermore, we failed to find any mutations in any of the potential off‐target sites (Figure 1c).

**Figure 1:**
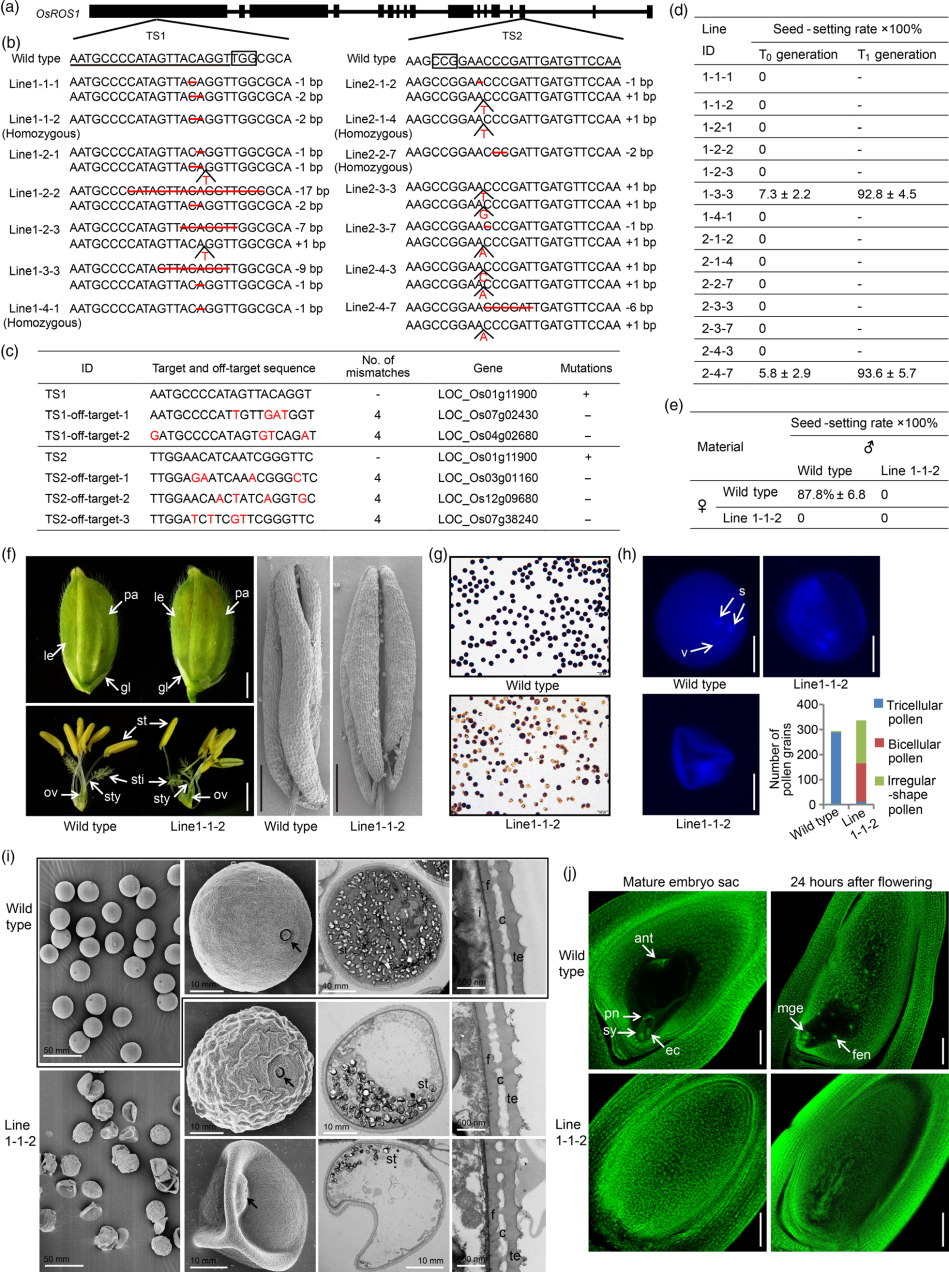
CRISPR/Cas9‐targeted mutagenesis of the *OsROS1* gene induces pollen and embryo sac defects in rice. (a) Sketch map of the target gene *OsROS1*. (b) The mutations of the knockout lines. The 20‐bp gene‐specific target sequences and PAM are underlined and boxed. The number of nucleotides deleted and/or inserted is indicated by the sign of minus (‐) and plus (+) followed by a number, respectively. (c) Off‐target effect examination. +, mutations detected; −, mutations not detected. (d) The seed‐setting rate of the *OsROS1* knockout lines. (e) The seed‐setting rate of the reciprocal crosses. ♀, crossed as female parents; ♂, crossed as male parents. (f) Comparisons of the appearance of spikelets (the left upper panel; bars = 2 mm), the spikelets after removing the lemma and palea (the left lower panel; bars = 2 mm), and SEM images of anthers (the right panel; bars = 0.5 mm). le, Lemma; pa, palea; gl, glume; st, stamen; sti, stigma; sty, style; ov, ovary. (g) I_2_‐KI staining of mature pollen grains. Bars = 100 μm. (h) DAPI staining results and the statistic of the tricellular, bicellular and irregular‐shaped pollen grains. s, sperm nuclei; v, vegetative nuclei. Bars = 25 μm. (i) SEM (the left two panels) and TEM (the right two panels) examination of the pollen grains. The most right panel are high magnification images of pollen wall. Arrows indicate the germination pore. st, starch granules; i, intine; f, foot layer; c, columella; te, tectum. (j) Comparison of the mature embryo sacs and the embryo sacs at 24 h after flowering. ant, antipodal cells; ec, egg cell; pn, polar nuclei; sy, synergid cells. mge, multi‐celled globular embryo; fen, free endosperm nuclei. Bars = 100 μm

During the vegetative growth stage, the growth state and appearance of all the mutant lines were indistinguishable from the wild type (WT); however, during the reproductive growth stage, most of the mutants were sterile. Of the 14 T_0_ mutants, only two produced seeds with a seed‐setting rate of 7.3 ± 2.2% (line 1‐3‐3) and 5.8 ± 2.9% (line 2‐4‐7), while the remaining 12 T_0_ frameshift mutants failed to produce any seed (Figure 1d). We analysed the nature of mutations by sequencing in the T_1_ generation of the two seed‐producing T_0_ mutants. We found that all the 11 T_1_ progeny of line 1‐3‐3 were homozygous mutants with the same 9‐bp deletion observed in the T_0_ plant, and that all the 9 T_1_ progeny of line 2‐4‐7 were homozygous mutants with the same 6‐bp deletion found in the T_0_ plant. The seed‐setting rates of these T_1_ mutants were comparable to that of the WT (Figure 1d). These results suggest that the transmission efficiencies of the mutant alleles were different. The in‐frame mutant alleles could be efficiently transmitted; while all of the editing events causing frameshift could not be passed on to the next generation. These results also showed that all the frameshifted mutants of TS1 and TS2 have the same effect on rice sterility determination. Combining the results of previous studies (Ono *et al.*, 2012), we speculated that the predominant function domain should be located in the C‐terminal of OsROS1.

To determine the underlying cause of the fertility defect of the gene editing mutant, we conducted hand‐pollinated reciprocal crosses between the ratoon plant of the homozygous mutant line 1‐1‐2 and the WT. When line 1‐1‐2 was used as the pollen receiver, the seed‐setting rate was zero; when the WT stigmas were sprinkled with the mutant pollen grains, the seed‐setting rate was zero as well. Only when WT stigmas were sprinkled with its own pollen grains, the seed‐setting rate was normal (Figure 1e). This finding indicates that the fertility defect of the *OsROS1* knockout mutants (e.g. line 1‐1‐2) is due to defects in both male and female gametophytes.

To dissect the cellular defects responsible for the sterility of the mutant, we compared development of the floret structure and gametophytes between line 1‐1‐2 and WT. The lemma, palea, glume, stamen, stigma, style and ovary of the mutant appeared to be normal, and after glume opening, the anther shape and dehiscence of the mutant were similar to that of the WT (Figure 1f). However, we observed difference in pollen fertility between the mutant and WT. I_2_‐KI staining showed that the pollens of the WT were regularly round and deeply stained; in contrast, the mutant pollens were irregular and slightly or not stained (Figure 1g). In addition, we also examined the tricellular pollens stained with DAPI and found that the WT pollen contained one dispersed vegetative nucleus and two smaller generative nuclei; however, in the mutant, about half of the pollen grains exhibited shaded and a bicellular feature, and the other half aborted showing irregularly shaped (Figure 1h). To further characterize the pollen difference between the WT and the mutant line 1‐1‐2, we examined the pollen grains using scanning electron microscopy (SEM) and transmission electron microscopy (TEM). Mature WT pollen grains were spherical and plump, containing large numbers of starch granules, and the wall of which composed of exine and intine; while in the mutant, the surface of about half of the pollen grains was wrinkled, and the other half had an irregular and shrunken appearance (Figure 1i). Furthermore, the pollen grains of both abnormal types accumulated little and abnormal starch granules and their wall lacked intine (Figure 1i). These findings suggest that the pollens of the *OsROS1* knockout mutants (e.g. line 1‐1‐2) are defective and aborted.

We further examined cytologically the embryo sacs. In the WT, the mature embryo sacs were plump, and the normal‐size sac cavities contained eight nuclei that could be recognized clearly, showing that antipodal cells located at the chalazal end, one egg cell and two synergid cells constituted the egg apparatus and located at the micropylar end, and two polar nuclei horizontally arranged above the egg apparatus; however, the embryo sacs were degenerated in the mutant, in which neither cavities nor nuclei could be observed (Figure 1j). At 24 h after pollination, when the double fertilization is completed in rice, almost all of the WT embryo sacs were normally fertilized each with a multi‐celled globular embryo and a layer of free endosperm nuclei; while in the mutant, although the degenerated sac cavities enlarged slightly, no fertilized egg was observed at all (Figure 1j). These findings suggest that the embryo sacs of the *OsROS1* knockout mutants (e.g. line1‐1‐2) are defective and aborted.

In summary, our results indicate that OsROs1 is essential for normal development of both male and female gametophytes and provide clues for further elucidating the biological mechanisms related to *OsROS1*‐mediated fertility regulation in rice. Moreover, we believe that the CRISPR/Cas9‐mediated genome editing technology would not like to be limited to *OsROS1* but applicable to investigating other genes with lethal effect on plant development.

## Conflict of interest

The authors have declared no conflict of interest.

## Author contributions

Y. X. and J. Y. designed the research; Y. X., F. W., Z. C., J. W., W. L., F. F., Y. T. and Y. J. performed the research; and Y. X. and Q‐H. Z. wrote the paper.
